# Spontaneous-Idiopathic Left Anterior Descending Artery Dissection: Is Watchful Waiting Better Than Immediate Stenting?

**DOI:** 10.1155/2013/639384

**Published:** 2013-09-10

**Authors:** A. Arrivi, M. Bazzucchi, M. De Paolis, A. Placanica, C. Bock, C. Milici, E. Boschetti, M. Dominici

**Affiliations:** Interventional Cardiology Unit, S. Maria University Hospital, Via Tristano Di Joannuccio 1, 05100 Terni, Italy

## Abstract

Spontaneous coronary artery dissection (SCAD) is a rare, complex disease, nowadays poorly understood yet. The lack of firm recommendations about this issue is a great limitation which makes any therapeutic decision controversial. The case described is that of a young, otherwise healthy woman, who presented with an ostial dissection of the left anterior descending (LAD) artery. Due to patient's stable clinical and hemodynamic parameters, we used a cautious approach based on watchful waiting and medical therapy, postponing stenting in order to achieve a partial vessel reopening with a more comfortable access to PCI.

## 1. The Case

A 46-year-old woman, with mild dyslipidemia and no prior cardiovascular events, was referred to our institute for a non-ST elevation-acute coronary syndrome (NSTE-ACS). Her past medical history was significant for anemia due to frequent spot bleedings from uterine leiomyoma.

At the admission, the patient had soft chest pain with normal clinical findings.

The ECG showed mild abnormalities of left ventricular repolarization, with normal left ventricular systolic function (LVEF) at the echocardiography. 

Laboratory exams during the hospitalization revealed both Troponin and CK-MB values' elevation (Troponin I 5.6 ng/mL; CK-MB 34.5 ng/mL). We also found moderate hypochromic and microcytic anemia (hemoglobin 9.1 g/dL; MCV 69.1 fl; MCH 21.1 pg).

We administered acetylsalicylic acid (250 mg i.v.), clopidogrel (600 mg loading dose), and heparin bolus (4.000 UI) followed by continuous infusion.

**Figure 1:**
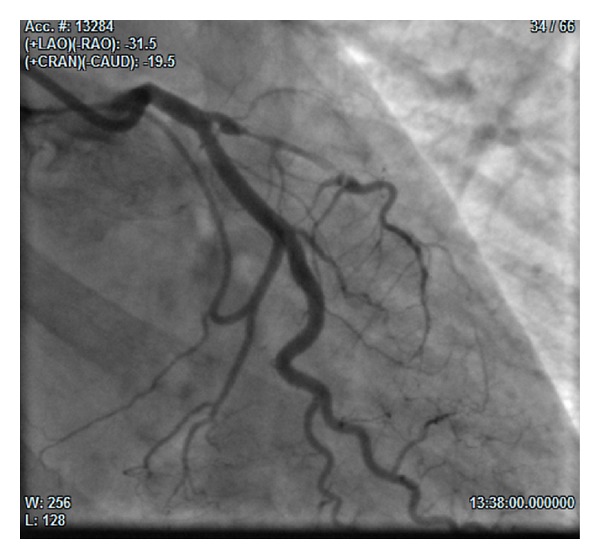
(31° RAO and 19° CAU): left anterior descending (LAD) ostial dissection, causing long proximal tract subocclusion and complete distal coronary occlusion.

The coronary angiogram, performed 24 hours after the admission, showed LAD ostial dissection, causing long proximal tract subocclusion and complete distal coronary occlusion (Figure 1). In consideration of the stable clinical conditions, no coronary interventions were performed. An intra-aortic balloon pump (IABP) was implanted, and the patient was transferred to the intensive care unit (ICU).

After gynaecological consulting, Decapeptyl was administrated to stop metrorrhagia, and martial therapy was started to correct anemia.

In the following 6 days the patient remained asymptomatic. No more episodes of metrorrhagia occurred, and hemoglobin value increased to 9.7 g/dL. 

We also observed decreasing values of Troponin and CK-MB.

**Figure 2:**
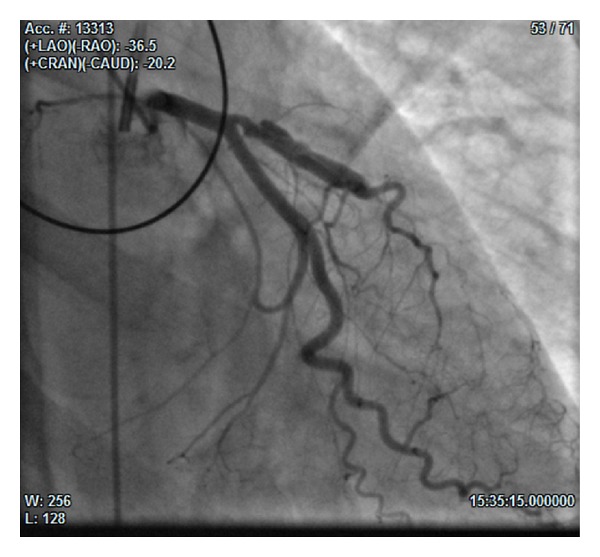
(36° RAO and 20° CAU): angiographic control at day 7 showing reopened dissection of the septalLAD and occlusion of diagonalLAD.

At day 7, an angiographic control showed reopened dissection of the septalLAD and occlusion of diagonalLAD (Figure 2). Even in this occasion we chose a conservative strategy, keeping the IABP on site (for other 10 days) without performing PTCA.

**Figure 3:**
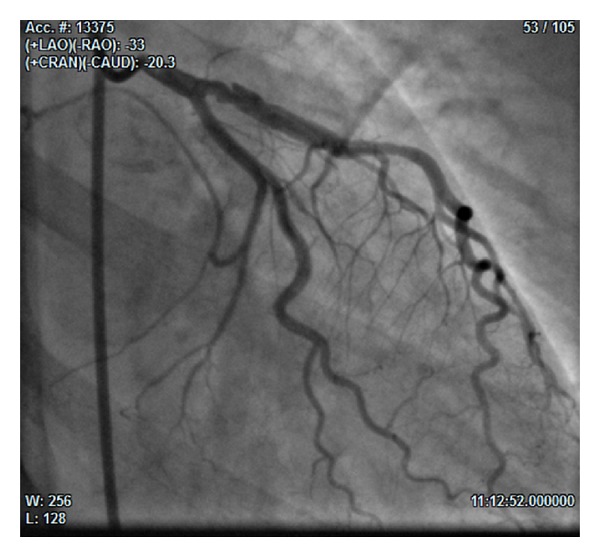
(33° RAO and 20° CAU): angiographic control at day 25 showing reopened LAD with TIMI 3 flow in the whole vessel.

**Figure 4:**
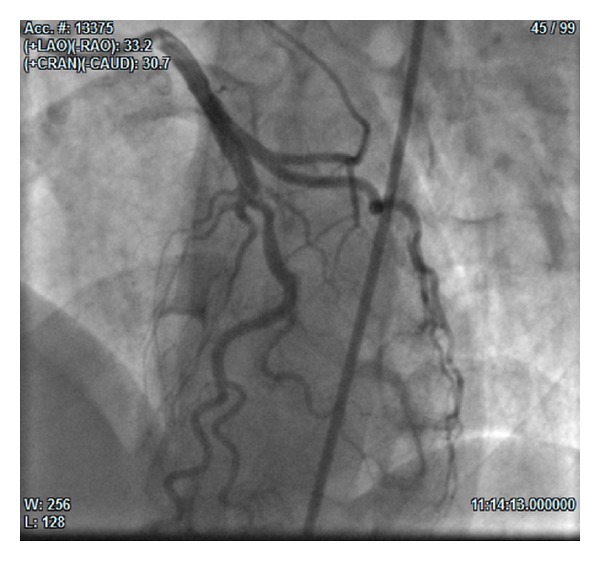
(33° LAO and 30° CRA): angiographic control at day 25 showing reopened diagonal-LAD.

**Figure 5:**
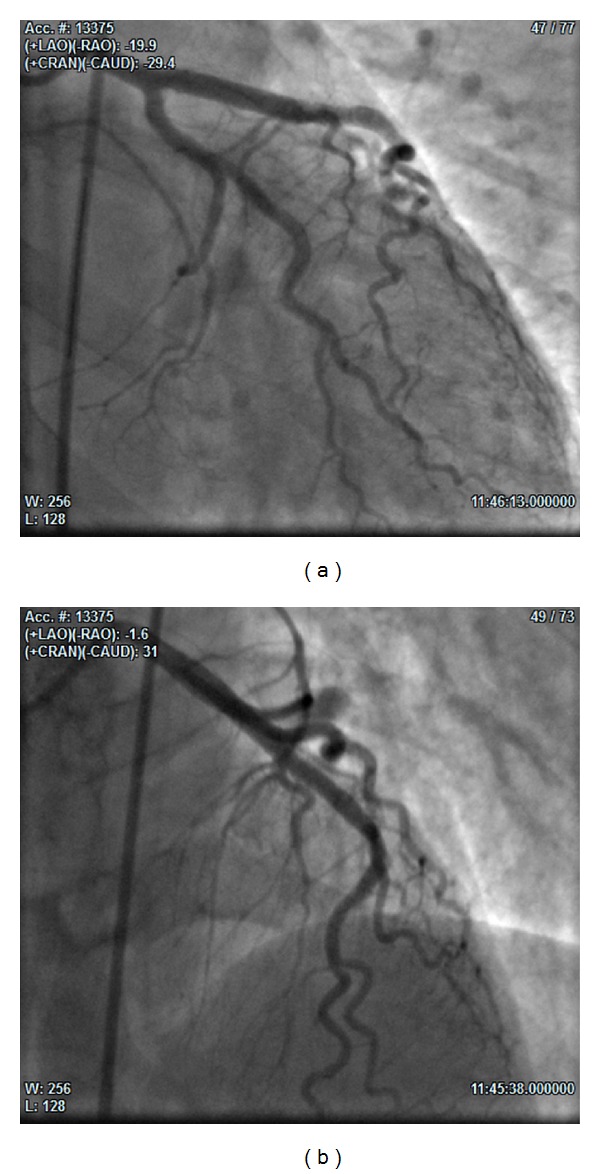
(a) (20° RAO and 29°CAU) and (b) (1° RAO and 31° CRA): final result after PCI with implantation of two DES in overlapping in the proximal-medium tract of LAD.

At the 3rd angiography (25 days from the admission), we found LAD dissection from the proximal to the medium tract, with spontaneous reopening of the diagonal branch and TIMI 3 flow in the whole vessel (Figures 3 and 4). On this occasion the operator performed PTCA with implantation of two 3.5 × 30 mm and 3.5 × 9 mm drug eluting stents (DES) in overlapping in the proximal-medium tract of LAD (Figure 5).

The patient remained free of clinic events in the following days: serial ECGs showed T waves inversion from V1 to V4. Laboratory research for autoimmunity diseases was negative. Cardiac enzymes decreased to normal values, and hemoglobin increased to 10.4 g/dL.

The patient was discharged 27 days after the admission with preserved LVEF at the echocardiogram. 

At the six-month followup, the patient was in a good clinical condition (NYHA I, CCS 0).

## 2. Discussion

Idiopathic coronary vessel dissection is a rare disease with an incidence of 0,1% among patients who undergo coronary angiography [1]. Most of the cases occur in young women with a predilection for the left coronary artery system [2]. At the presentation the mortality rate is high [3], with a progressive decrease during the subacute phase [4]. The etiology and pathogenesis of SCAD are not clearly understood yet. Changes within the arterial wall and/or abnormal shear forces are implicated. Atherosclerotic plaque inflammation and rupture may cause disruption of the intimal-medial junction, resulting in an intimal flap and subsequent intramural hematoma formation. Hemorrhage from weakened vasa vasorum in the outer tunica media leading to compression of the lumen and subsequent dissection has been proposed as another possible mechanism leading to SCAD [5]. The clinical presentation ranges from unstable angina to sudden cardiac death, depending on the extent and severity of the dissection [6].

 The lack of firm recommendations about this issue is a great limitation which makes any therapeutic decisions controversial. The treatment options for SCAD include medical treatment, percutaneous coronary intervention with stenting, and bypass graft operation [7].

Nowadays, clinical purposes are based on the operator's personal experience and the literature's reports [8].

The case described is that of an otherwise healthy woman who presented with NSTE-ACS due to proximal LAD dissection. Despite this dramatic angiographic picture, the patient was asymptomatic with stable hemodynamic parameters. So, we initially decided to use a cautious approach based on watchful waiting, IABP implantation, and medical therapy. Due to the patient's lasting clinical conditions at day 7, we did not perform PTCA in order to obtain a spontaneous reopening of the diagonal-LAD, as already occurred with the septal-LAD. PCI with stenting was performed only after 25 days, when angiography showed spontaneous reopening of the whole LAD. This finding helped the operator to better define the dissection features, allowing a more simple and safe approach to the PCI procedure, strictly limiting the stenting to the injured tract of the vessel. Indeed, technical issues during PCI include placing the guidewire in the true lumen rather than the dissection plane and ensuring sealing of the dissection entry point with an appropriately sized stent [9]. An important limitation of a too much early PCI is represented by the risk of coronary perforation in the case of entry and passage of the guidewire into the false lumen, as well as the need of stenting long coronary tracts and bifurcations. We believe that a conservative approach, when clinically possible, should be initially preferred to any interventional treatment. The good clinical condition as well as the preserved LVEF at the followup justified a posteriori the cautious strategy used in this case.

## Abbreviations

LAO: Left anterior oblique
RAO: Right anterior oblique
CRA: Cranial
CAU: Caudal.
